# Performance of a New Al_2_O_3_/Ce–TZP Ceramic Nanocomposite Dental Implant: A Pilot Study in Dogs

**DOI:** 10.3390/ma10060614

**Published:** 2017-06-03

**Authors:** Roberto Lopez-Píriz, Adolfo Fernández, Lidia Goyos-Ball, Sergio Rivera, Luis A. Díaz, Manuel Fernández-Domínguez, Catuxa Prado, José S. Moya, Ramón Torrecillas

**Affiliations:** 1 Nanomaterials and Nanotechnology Research Centre (CINN), CSIC-University of Oviedo (UO), Avda. de la Vega 4-6, El Entrego, San-Martín del Rey Aurelio 33940, Spain; a.fernandez@cinn.es (A.F.); la.diaz@cinn.es (L.A.D.); c.prado@cinn.es (C.P.); jsmoya@icmm.csic.es (J.S.M.); r.torrecillas@cinn.es (R.T.); 2 Nanoker Research, Pol. Ind. Olloniego, Parcela 22A, Nave 5, Oviedo 33660, Spain; l.goyos@cinn.es (L.G.-B.); s.rivera@nanoker.com (S.R.); 3 Maxillofacial Surgery Department, Madrid-Montepríncipe Hospital, CEU-San Pablo University, Madrid 28003, Spain; clinferfun@yahoo.es

**Keywords:** ceramic implant, nanocomposite, Ce-TZP, Al-TZP, zirconia implants, in vivo study

## Abstract

Although titanium remains as the prevalent material in dental implant manufacturing new zirconia-based materials that overcome the major drawbacks of the standard 3Y-yttria partially-stabilized zirconia (Y-TZP) are now emerging. In this study, a new ceramic nanocomposite made of alumina and ceria-stabilized TZP (ZCe-A) has been used to produce dental implants with the mechanic and topographic characteristics of a pilot implant design to evaluate bone and soft tissue integration in a dog model (*n* = 5). Histological cross-section analysis of the implanted ceramic fixations (*n* = 15) showed not only perfect biocompatibility, but also a high rate of osseous integration (defined as the percentage of bone to implant contact) and soft tissue attachment. This clinical success, in combination with the superior mechanical properties achieved by this Al_2_O_3_/Ce-TZP nanocomposite, may place this material as an improved alternative of traditional 3Y-TZP dental implants.

## 1. Introduction

Dental implants are considered an essential treatment modality for the replacement of missing teeth. There is a great number of data proving the significant and predictable performance of implants in partially- and totally-edentulous arches in the long-term. However, the aesthetic outcome of implants placed in the anterior zone is challenging [1,2].

The success or failure of dental implants in vivo critically depends on the biological (molecule, cell, and tissue) interactions at the implant/tissue interface. It is well known that, depending on the particular functionality of the different material surfaces present in a dental implant, the chemical, mechanical, topographic, and electrical material properties will undoubtedly contribute to the performance of a biomaterial/prosthetic device. This is why the development of new mechanically-improved ceramic materials for dental implant applications have to be coupled with a deep material/tissue interaction study.

Titanium has been the primary choice of manufacturers because of the biocompatibility, favourable bone and soft tissue response, and adequate mechanical strength and corrosion resistance of commercially-pure titanium (cpTi) and Ti-6Al-4V alloys (Ti64).

The clinical effectiveness and the osseous integration of titanium implants have been documented; the success of implants in partially-edentulous patients is located between 96.6% and 98.5%. However, regarding aesthetic demands, the complete implantation of a tooth is still a challenge, since success does not only depend on the implant’s osseous integration and functionality, but also on the harmonious integration of the crown in the dental arc. In the case of especially delicate zones, from an aesthetic point of view, as could be the case of patients with a high line of smile, exigencies will be at a maximum, including the optimal positioning of the implants. 

In spite of the numerous improvements in the design and manufacture of metallic implants and abutments, there is still a risk of the metallic components of implants being visible. Even when implants are subgingival, titanium’s grey colour can cause the tissue to adopt an unnatural bluish colour. This can be attributed to the slimness of the gingival tissue surrounding the abutment, which is insufficient to block the reflection of the light from the metal to the surface. 

There are other drawbacks associated with titanium implants; there is an increasing concern about the potentially hazardous impact of titanium implants on systemic health. First-generation titanium alloys, represented by Ti64, have been reported to cause toxicity and be connected to allergic reactions. In addition, elevated concentrations of titanium have been found in the vicinity of oral implants, regional lymph nodes, and serum [3,4]. However, the clinical significance of these data remains unclear, but it is a fact that these concerns have rendered many patients to seek a metal-free solution [5,6].

To reach optimal mucogingival aesthetics, it has been necessary to use ceramic materials to manufacture both implants and abutments. Up to now ceramic implants have been made of two types of materials: high-purity alumina (Al_2_O_3_), introduced by Sandhaus in 1987 as Cerasand^®^ (Lausanne, Switzerland), and yttria partially-stabilized zirconia (Y-TZP), which has been widely adopted for ceramic endosseous implants, implant abutments and all-ceramic crowns. Y-TZP combines good mechanical, tribological, and biological qualities with a white colour and, as a result, has been introduced by several implant manufacturers. Nevertheless, there is very large concern about the long-term durability of Y-TZP due to the aging or low temperature degradation (LTD) of 3Y-TZP, which basically involves a phase transformation that leads to microcracking, resulting in catastrophic failures which have been well documented in in vivo reports [7,8,9,10]. Both materials present good optical and mechanical properties, although they show important differences in terms of microstructure and effectiveness against defect propagation. On one hand, Y-TZP’s fracture resistance is twice that of Al_2_O_3_; however, Y-TZP can suffer LTD. On the other hand, the colour of Al_2_O_3_ is very close to that of natural teeth, so Al_2_O_3_ implants or abutments hold certain advantages over Y-TZP from an aesthetic point of view. In this sense, over previous years, coloured zirconia has been developed to fulfill aesthetic parameters. Therefore, it is necessary to develop new ceramic materials that conjugate the required mechanical and aesthetic performances. Furthermore, there is strong evidence that proves ceramic materials are less prone to plaque accumulation than metal substrates [11] and it is well known that peri-implantitis is a major concern in terms of implant long-term survival [12]. 

The aforementioned reasons have prompted the research of new ceramic nanocomposite materials that overcome the major drawbacks of the standard 3Y-TZP that is currently available in the dental market [13,14].

The moderate toughness and the inherent drawbacks of zirconia lead to an increasing interest in alumina-zirconia composites and/or nanocomposites as potential ceramics for biomedical applications. Some authors have developed zirconia-doped alumina ceramics to improve the structural behaviour of monolithic alumina [15,16,17,18,19,20].

De Aza et al. [21] studied different compositions with different zirconia additions, showing a clear improvement in mechanical properties without ageing. Some authors [22] performed ageing studies on the alumina-zirconia system showing that 3Y-TZP-alumina composites above 16 vol % zirconia show significant ageing related to the percolation threshold, above which a continuous path of zirconia grains allows transformation to proceed.

ZTA composite materials can be obtained through different processing routes. Conventional methods include the mechanical mixing of powders following different milling systems, including attrition and ball milling [23,24].

Other authors [25,26] have suggested using ceria-stabilized tetragonal zirconia polycrystals (Ce-TZP) as a second phase to improve the toughness of alumina composites. Ce-TZP is well known as a ceramic with high toughness and high resistance to low-temperature thermal degradation and, therefore, the use of Ce-TZP in alumina-zirconia materials is expected to significantly increase their fracture toughness. Moreover, during recent work, the good mechanical and biological behaviour of 3D-printed scaffolds prepared from this nanocomposite was proved [27].

In this pilot work, we study the suitability of an Al_2_O_3_/Ce-TZP ceramic nanocomposite for dental implant applications in terms of osseous integration, defined as the percentage of bone to implant contact and soft tissue attachment.

## 2. Materials and Methods

Samples were prepared in the form of discs (for surface roughness characterization and in vitro studies), standardized samples (for mechanical tests), and implant-shaped bodies (for surface roughness characterization and in vivo studies).

### 2.1. Powder Processing and Microstructural Characterization

The initial precursor powders used to manufacture the material are described in Table 1.

These powders were mixed in a proportion of 34.5/65.5 vol % (Al_2_O_3_/ZrO_2_) by attrition milling for 4 h using isopropanol as the solvent and a solid content of 70%. A deflocculant agent based on a carboxylic acid was added to the slurry (0.35 wt %) and a plasticizer based on carbohydrates was also added (0.2 wt %). The slurry was poured into a cylindrical plaster mould (diameter: 25 mm, height: 125 mm) following a slip casting process. The resulting bar was dried for four days in an oven at 60 °C. This bar was then inserted into a polyurethane mould and subjected to cold isostatic pressing up to 200 MPa (2000 bar) for final compaction. Subsequently, the pressed bar was pre-sintered in an atmospheric furnace up to 950 °C at a low heating rate (0.5 °C/min) to remove organics. The pre-sintered bar was then machined with diamond tools in a lathe to obtain the final implant shape and, finally, the implant was sintered in an atmospheric furnace at 1450 °C for 2 h.

### 2.2. Characterization of the Alumina/Ce-TZP Nanocomposite

#### 2.2.1. Mechanical Properties of Alumina/Ce-TZP Material

The bending strength σ_f_ was determined via the four-point bending test using prismatic bars of 4 mm width, 40 mm length, and 3 mm thickness. The tensile surface of the bars was polished down to 1 µm. The tests were performed at room temperature using a universal testing machine (Instron Model E10000, Boston, MA, USA). The specimens were loaded to failure with a cross-head speed of 1 mm/min and a span of 12.5 mm according to ISO 6872:2008 standard. Reported strengths represent the mean and standard deviation of at least five specimens.

Fracture toughness (*K*_1c_) was measured using single edge notched beams (SENB, dimensions: 3.0 × 4.0 × 45 mm^3^). The tests were performed at room temperature using the same testing machine as for flexural strength determination, at a crosshead speed of 0.5 mm/min with a span of 40 mm. Notches were introduced with a diamond blade saw.

The microstructural characterization of the surfaces, which were polished down to 1 µm and then thermally etched (1350 °C, 5 min), was performed using scanning electron microscopy (FEI Quanta 650 FESEM (Field Emission Scanning Elentron Microscopy), Eindhoven, The Netherlands).

#### 2.2.2. Determination of Surface Topography (Discs and Implants)

Surface roughness (*R*_a_), defined as the arithmetic average of the profile ordinates within the measured section (average height), was measured according to DIN EN ISO 4287. A Leica TCS SP2 Spectral Confocal and Multiphoton System was used for image acquisition applying a krypton/argon 488 nm laser and a 10×/0.40 NA objective (PL APO 10×/0.40 CS). Leica’s Quantimet Quantitative Metallography Image Analysis Software was used for image analysis and data extraction. The *R*_a_ of each ceramic disc represents the average of five random measurements. The *R*_a_ of the ceramic implants was measured before implantation in terms of peak and valley roughness; three different peaks and three different valleys were randomly selected and profiled. Peak 1 and valley 1 are, out of the selected sections, the closest to the abutment, while peak 3 and valley 3 are, out of the selected sections, are the furthest away from the abutment. Their specific *R*_a_ is the average of three random measurements. In both cases, the region of interest (ROI) or measurement area corresponds to 2000 µm^2^.

### 2.3. Determination of the In Vitro Response

#### 2.3.1. Cell Immunostaining and Imaging

SAOS-2 (SArcoma OSteogenic; human primary osteosarcoma osteoblasts of epithelial morphology) cells were incubated on sterilized ceramic discs in 48-well plates at a concentration of approximately 3 × 10^4^ cells/well for 48 h at 37 °C in a humidified 5% CO_2_ atmosphere. Standard cell culture medium DMEM (GIBCO^®^, Invitrogen, Carlsbad, CA, USA) was applied; it was not supplemented with osteogenic factors. After incubation, different proteins related to osteoblast differentiation (SPARC, or osteonectin, and transcription factor RUNX2) were labelled with antibodies (primary antibodies: mouse anti-human IgG1 SPARC and goat anti-human IgG RUNX2, from R and D Systems, and secondary antibodies: rabbit anti-mouse IgG1 conjugated with Alexa fluor 488 and rabbit anti-goat IgG conjugated with Alexa fluor 555, from Invitrogen). DAPI, a fluorescent blue dye, was also used for DNA marking. The stained cells were visualized and imaged using a Leica TCS SP2 Spectral Confocal and Multiphoton System.

#### 2.3.2. In Vitro Cytotoxicity

SAOS-2 cells were seeded onto sterilized material samples in 48-well plates at a density of approximately 1 × 10^4^ cells/mL. Empty wells, seeded with the same amount of cells, were used as controls (blanks). Cellular viability was determined after 48 h of incubation in DMEM (GIBCO^®^, Invitrogen, Carlsbad, CA, USA) at 37 °C in a humidified 5% CO_2_ atmosphere using the CellTiter 96^®^ AQueous One Solution Cell Proliferation Assay (Promega, Madison, WI, USA). Absorbance (Abs) was determined at 490 nm using a BIO-RAD Model 680 Microplate Reader and the percent cell viability was calculated as follows:

% Viability = 100 × Abs_sample_/Abs_blank_

All assays were conducted in triplicate.

#### 2.3.3. In Vitro Osseous Differentiation Determination

In vitro tests were performed on three different materials: a novel ceramic nanocomposite (ZCe-A), a material renowned for its biocompatibility (SPA-05 alumina), and the dental ceramic gold-standard (3Y-TZP). All three materials were studied in the shape of discs: (i) polished and (ii) grit-blasted until surface roughness (*R*_a_) values reached 1.0–1.5 μm.

The osseous differentiation ability of the cells cultured on the ceramic discs was evaluated by quantification of alkaline phosphatase (ALP) activity. SAOS-2 cells were seeded onto sterilized substrates in 48-well plates at a density of approximately 2 × 10^4^ cells/mL. Empty wells, seeded with the same amount of cells, were used as controls. The cells were incubated for seven days at 37 °C in a humidified 5% CO_2_ atmosphere. Supplemented culture medium was used and replaced every 2–3 days. The osteogenic factors that were added to the standard cell culture medium, DMEM (GIBCO^®^, Invitrogen, Carlsbad, CA, USA), were: ascorbic acid (0.2 mM, final concentration), β-glycerophosphate (10 mM, final concentration), and dexamethasone (0.1 μM, final concentration), all from Sigma Aldrich (St. Louis, MS, USA). ALP activity was determined using the SensoLyte^®^ pNPP alkaline phosphatase assay kit (AnaSpec Inc., Fremont, CA, USA) and absorbance was measured at 405 nm on a BIO-RAD Model 680 microplate reader. All assays were conducted in triplicate.

### 2.4. In Vivo Test

#### 2.4.1. Animals

The Ethics Committee for Animal Research Welfare approved the study protocol to be carried out at the Minimally Invasive Surgery Center, in Cáceres, Spain. Five four-year-old Beagle dogs were used. Sample size was calculated taking ethical considerations and the sample sizes used in similar studies into account. Veterinary assistance was mandatory during all procedures. General anaesthesia was induced with intravenous injected propofol 10 mg/kg (Propofol Hospira, Hospira Productos Farmacéuticos y Hospitalarios, Madrid, Spain). One no. 7 endotracheal tube with a balloon cuff was placed and connected to a circular anaesthesia circuit (Leon Plus, Heinen and Löwenstein, Bad Ems, Germany). The anaesthesia was sustained with sevofluorane (Sevorane, Abbott Laboratories, Madrid, Spain). Multimodal analgesia was employed in the perioperative (ketorolac 1 mg/kg (Toradol 30 mg, Roche), tramadol 1.7 mg/kg (Adolonta inyec., Grünenthal) and buprenorfine 0.01 mg/kg (Buprex, Reckitt Benckiser Pharmaceuticals Limited, Berkshire, UK)).

#### 2.4.2. Surgery

All mandibular and maxillary first molars were extracted from five male four-year-old Beagle dogs. After two months of healing, mucoperiosteal flaps were elevated and osteotomy preparation of the implant beds was performed according to a conventional increasing diameter drilling sequence up to a twist drill of 3.20 mm diameter with external irrigation (Seven Surgical Kit MK-0037, MIS Dental Implants, Savion, Israel). For bone densities of types 3 and 4 a countersink for a standard platform was used to avoid overcoming an implant torque insertion of 50 Nw. For implant placement, a short insertion tool for internal hexagon connection was directly applied to the implant. Three nanocomposite Al_2_O_3_/Ce-TZP implants of 4 mm in diameter and 10 mm in length (threaded section) were inserted per dog, two in the mandibular first molar position and one in the maxillary first molar position. Implant allocation was performed randomly.

#### 2.4.3. Histological Preparation and Analysis

After eight weeks of implantation the animals were euthanized with a lethal dose of sodium pentothal. Mandibular blocks containing fixtures were retrieved and stored in a 5% formaldehyde solution (pH 7). The implant blocks were retrieved from the jaw bone using an oscillating autopsy saw (Exakt, Kulzer, Germany). The dissected specimens were immediately immersed in a solution of 4% formaldehyde and 1% calcium and processed for ground sectioning following the Donath and Breuner method [18]. Each implant block was individualized, embedded in methyl methacrylate, and stained with combined Harris Haematoxyline and Wheatley. The histological analysis was performed by using a transmitted light microscope (Optiphot 2-POL, Nikon, Japan) equipped with a digital camera (DP-12, Olympus, Japan).

The images obtained were processed with ImageJ version 1.46 r software (Rasband, 2012). Morphometric readings were performed on at least three preparations per defect. When necessary, a polarized light microscope was employed to determine the boundaries of the newly-formed bone. All measurements were taken by the same researcher, and boundaries were revised by a second one. To determine the reproducibility and measurement error, ten randomly-selected slides were measured three times on three different days.

## 3. Results and Discussion

### 3.1. Nanocomposite Characterization

**Figure 1:**
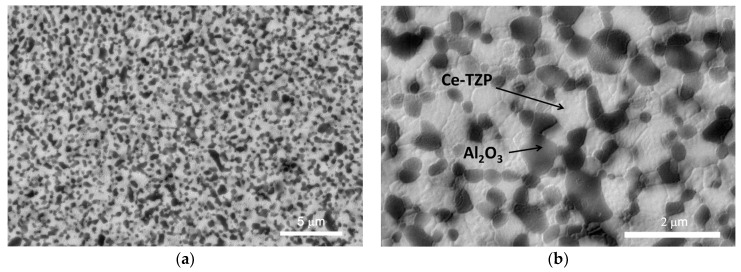
SEM micrographs of the ZCe-A nanocomposite at different magnification. (**a**) 10,000× and (**b**) 40,000×.

The Al_2_O_3_/Ce-TZP nanocomposite was characterized before its implantation in vivo. Figure 1 shows FESEM images of the material’s microstructure. Figure 1a (low magnification) shows the general microstructure of the nanocomposite which consists of a good dispersion of round alumina particles with a mean grain size of about 400 nm (dark grey phase) in a Ce-TZP matrix, formed by crystals of about 500 nm. Alumina crystals are located mainly at intergranular positions although some alumina crystals can be observed at intragranular positions as well. No pores or agglomerates can be observed, which implies a fully dense material. 

The flexural strength and fracture toughness of the ZCe-A nanocomposite material were calculated to be 910 ± 30 MPa and 9.1 ± 0.2 MPam^1/2^, respectively. In comparison to commercial Y-TZP, the fracture toughness of ZCe-A is almost two times higher while their mechanical strength is very similar. Additionally, there is no risk of ageing (degradation at low temperature) for ZCe-A [28], which is one of the main limitations for using Y-TZP in implantology.

### 3.2. In Vitro Biological Assays

3Y-TZP and alumina were selected as the reference materials to evaluate the in vitro performance of ZCe-A, the alumina, and the Ce-TZP composite material being studied in this work. 3Y-TZP was selected instead of Ce-TZP because the former is the gold-standard ceramic material for dental applications while the latter is not used in this field as a monolithic material.

SAOS-2 cells (human primary osteogenic osteosarcoma osteoblasts) were selected for this study because they are the most investigated and used cell line for studying the suitability of biomaterials for bone contact or as bone substitutes.

ISO 10993-5 states that a material is considered non-cytotoxic when cell viability exceeds 70%. In this case, all of the studied discs allowed for almost 100% cell viability and, therefore, none of them can be considered cytotoxic.

**Figure 2:**
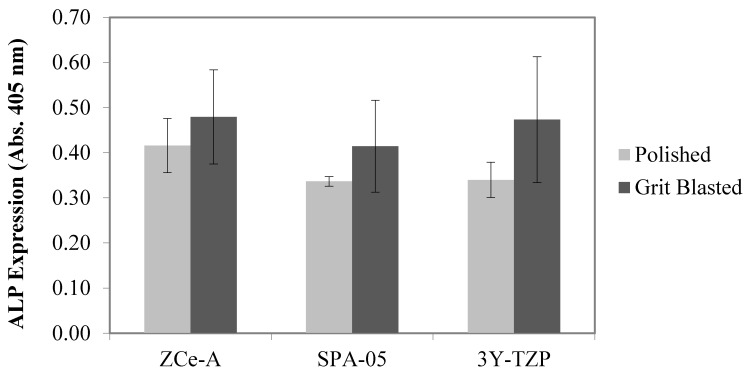
ALP expression (absorbance at 405 nm) achieved on polished and grit-blasted discs after incubation of SAOS-2 for seven days. The error bars represent standard deviation values.

ALP levels increase when active bone formation (osseous differentiation) occurs, as it is a by-product of this process. pNPP (*p*-nitrophenyl phosphate) is a chromogenic substrate for ALP and can be used to detect its activity in biological samples. Upon dephosphorylation, pNPP turns yellow and can be detected by measuring absorbance at 405 nm. Therefore, the amount of osseous differentiation achieved on our discs was evaluated by measuring sample absorbance at 405 nm after the enzymatic reaction with pNPP. The level of ALP activity present on the polished and grit-blasted ceramic discs after seven days of incubation is shown in Figure 2. We can state that ZCe-A and 3Y-TZP induce the most osseous differentiation and the grit-blasted surfaces are more effective than the polished surfaces in every case.

After achieving quantitative results concerning osseous differentiation on our ceramic discs, we decided to perform a more qualitative analysis. For this purpose, after cell incubation, different osteogenic differentiation markers (SPARC, or osteonectin, and RUNX2) were labelled with specific antibodies and fluorescent molecules and imaged using a confocal microscope.

**Figure 3:**
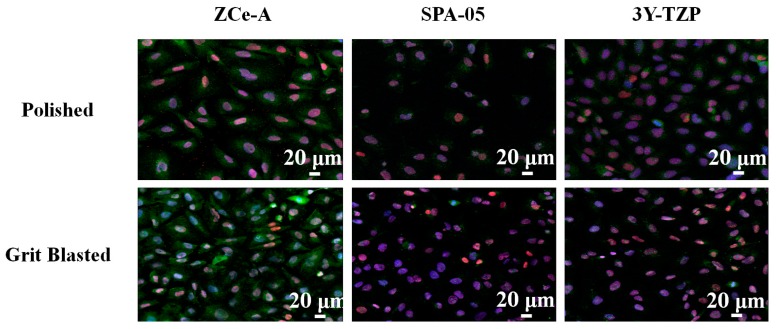
SPARC (green)—RUNX2 (red)—DAPI (blue) staining of SAOS-2 cells incubated for 48 h on the surface of polished and grit-blasted ZCe-A, SPA-05, and 3Y-TZP samples.

In Figure 3, the top three images show the expression of the targeted molecules on the surfaces of polished ZCe-A, SPA-05, and 3Y-TZP samples, respectively. In turn, the bottom three images show the expression of the labelled molecules on the surfaces of grit-blasted ZCe-A, SPA-05, and 3Y-TZP discs, respectively. In both cases we can see that on ZCe-A polished samples cells are more widespread and show very good osteogenic marker expression. On both SPA-05 and 3Y-TZP there is less expression. The larger number of cells present on the grit-blasted samples implies that more cellular proliferation occurs on the rougher surfaces, especially on ZCe-A. Furthermore, in the case of ZCe-A, we can appreciate increased SPARC (green) expression in the cytoplasm, while this is not reached either in the case of SPA-05 or 3Y-TZP. In summary, we can appreciate both a composition effect and a roughness effect, where ZCe-A is the most favourable material and the grit-blasted surface is more favourable than the polished surface for cellular osteogenic differentiation. 

Once the positive influence of ZCe-A’s composition and roughness had been proved, 15 dental implants were manufactured out of this material following the procedure described in Section 2.1. Table 2 shows the roughness values that were obtained.

**Figure 4:**
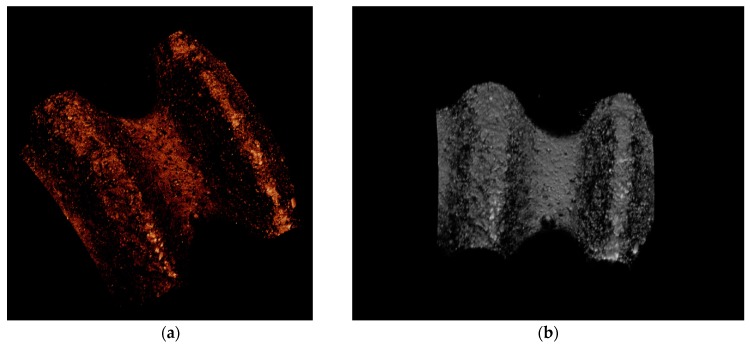
3D profiles of a ZCE-A implant surface at different angles. (**a**) oblique view and (**b**) front view.

The average peak and valley surface roughness and standard deviation values were found to be *R*_a peak_ = 1.38 ± 0.24 µm and *R*_a valley_ = 0.93 ± 0.10 µm, respectively. Thus, the measured roughness values agree with those that were proven as beneficial for cell response during in vitro tests [29]. Figure 4 shows 3D profiles of the implant’s surface.

The material’s surface topography is regarded as decisive in terms of tissue response. Matching material surface properties and biological processes is a key point when engineering surface features to obtain desired biological reactions. The results of this study are in accordance with those of in vivo studies that have demonstrated a better performance of moderately rough (*R*_a_ 1.0–2.0 µm) implants, in comparison to minimally rough implants (*R*_a_ 0.5–1.0 µm), highly rough implants (*R*_a_ > 2 µm), or turned implants [30]. Furthermore, inflammatory cascade systems are triggered by material-surface interactions. A modulated inflammatory phase during osseointegration is a key point to avoid inducing deleterious bone reposition and osseointegration failure. Data indicate that inflammatory cells respond to surface texture and, thus, the expression and secretion of proinflammatory cytokines is increased on rough surfaces when compared to relatively smooth surfaces [31]. The surface roughness of the Al_2_O_3_/Ce-TZP nanocomposite implants corresponds to a moderate roughness that is associated with a promotion and downregulation of leukocyte accumulation, adhesion, and secretion of proinflammatory substances.

### 3.3. Histological Descriptive Interpretation of Thin Ground Preparations.

**Figure 5:**
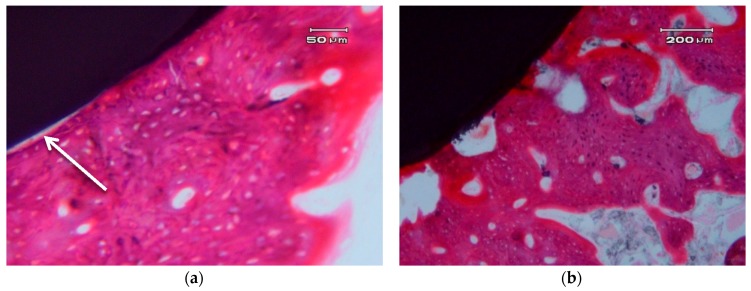
Transversal cross-sections of different nanocomposite ZCe-A implant-bone interfaces after eight weeks of healing. (**a**,**b**) show two different interfaces.

Figure 5 shows the photographs correspond to a transversal cross-section of implant.

In general, we can clearly appreciate bone growth in direct contact with the surface of the implant. There are some areas that present a small gap of less than 5 µm, which can be due to an artefact of the histological process (see arrow in Figure 5a). There is no connective tissue interposed between the surface of the implant and the bone. There are no signs of inflammation at the interface or of adjacent bone. The bone that is in contact with the implant shows, in some areas, very few characteristics of immature bone and a typical woven bone cell distribution (plexiform) (Figure 5b), although it mostly consists of laminar and haversian bone. There are no images of bone sequestration, nor atypia or dysplasia.

**Figure 6:**
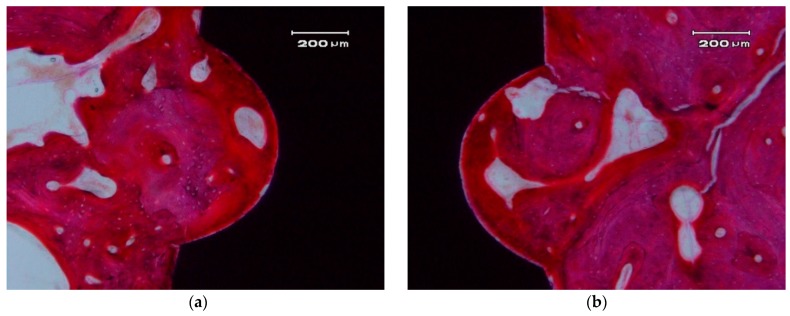
Bone apposition on the surface of a ZCe-A implant after eight weeks of healing (longitudinal cross-section). The bone in contact with the implant shows a lamellar and Haversian structure. (**a**) left side and (**b**) right side of the implant

**Figure 7:**
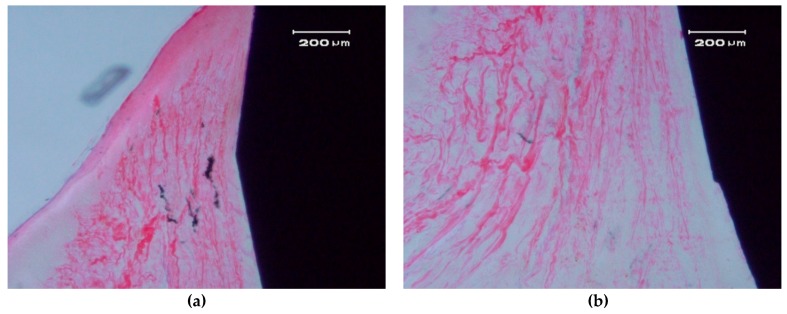
Contact between a ZCe-A nanocomposite implant and soft gingival peri-implant tissue after eight weeks of healing (longitudinal cross-section). (**a**,**b**) show two different interfaces.

Figure 6 and Figure 7 show longitudinal sections of the implant-bone interface. 

In the hard tissue area, a direct and close apposition of the bone matrix to the implant surface is observed, without the presence of a non-mineralized connective tissue interface. 

The soft, gingival, peri-implant tissue is found to be in perfect contact with the implant. There is a close epithelial adhesion to the implant surface (Figure 7a) without lymphocytic infiltrate, disruption of the keratinized gingival structure, or alterations of the connective tissue attachment (Figure 7b). Secondary and tertiary osteons and vascular buds are seen nearby the implant’s surface (Figure 7a). Bone grows inside the thread’s valleys in intimate contact with the implant and very clear reversal lines can be observed delineating the different stages of bone remodeling (Figure 7b). There are no signs of inflammation or implant rejection by the bone, nor atypia or dysplasia.

**Figure 8:**
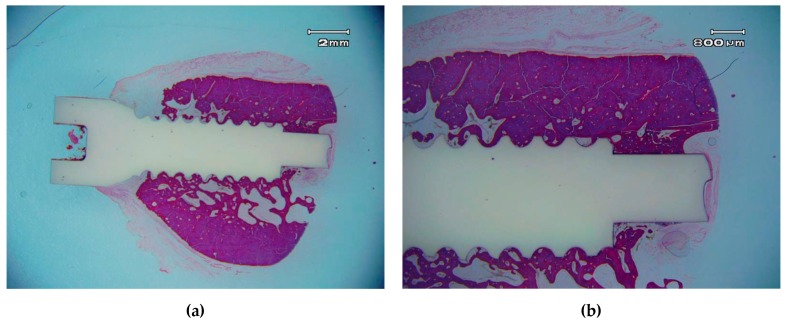
Osseo-integrated ZCe-A nanocomposite implant after eight weeks of implantation in the posterior maxilla of a Beagle dog at different magnifications. (**a**) scale bar 2 mm and (**b**) scale bar 800 µm.

ZCe-A nanocomposite implants showed high bone-to-implant contact (BIC) values (80 ± 5% after eight weeks) (Figure 8). These values are similar to those found by Saulacic et al. [32] for commercially-pure titanium implants (85% BIC) and titanium-zirconia implants (72% BIC), and quite a lot higher than those reported for Ti64 implants (30% BIC). In another recent study performed in miniature pigs [33], mean BIC values of zirconia implants differ depending on the surface modification method applied: sand-blasting (30% BIC), sand-blasting and acid-etching (48% BIC), and sand-blasting and alkaline-etching (22% BIC). Despite the different animal models used, these zirconia implant BIC values are significantly inferior to the osseous integration rate achieved by the ZCe-A nanocomposite, as shown in Figure 8 in posterior maxillary bone. Furthermore, we have not found multinucleated giant cells (MNGCs) along the implant surface in accordance with data found for cpTi implants (0% MNGCs) and in contrast with data found for TiZr implants (3% MNGCs), Ti64 implants (50% MNGCs), sand-blasted Zr implants (17% MNGCs), sand-blasted and acid-etched Zr implants (38% MNGCs), and sand-blasted and alkaline-etched Zr implants (41% MNGCs).

## 4. Conclusions

All of the materials (ZCe-A, alumina and YTZP) and surface formats (polished and grit-blasted) tested in vitro proved to be biocompatible. ZCe-A proved to be the most favourable material for osseous differentiation in vitro as it induced the fastest and most osteogenic gene expression. Following the in vitro assays, ZCe-A dental implants were placed in a superior animal model in vivo, showing bone-to-implant contact (BIC) values of 80 ± 5% after eight weeks, similar to those reported for cpTi and TiZr implants, and much higher than those reported for Zr implants. Furthermore, the ZCe-A implants attached closely and firmly to the surrounding soft tissues and showed no signs of inflammation. Despite the inherent limitations of a pilot study performed in an animal model, the performance of the ZCe-A dental implants in vivo led them to be conceived as a real alternative to titanium and traditional zirconia implants.

## Figures and Tables

**Table 1:** Description of starting powders.

Material	Supplier	Designation	Purity	d_50_ (nm)	Specific Surface
**Al_2_O_3_**	Sasol	SPA 0.5	>99.9%	380	7.7 m^2^/g
**ZrO_2_ 10% CeO_2_**	Daiichi Kigenso	10 Ce-TZP	>99.9%	35	14.3 m^2^/g

**Table 2:** Surface roughness values of different peaks and valleys that were randomly selected along the ceramic implant.

Parameter	*R*_a_ 1 (µm)	*R*_a_ 2 (µm)	*R*_a_ 3 (µm)	Avg. *R*_a_ (µm)	*R*_a_ S.D. (µm)	Overall Avg. *R*_a_ (µm)	Overall *R*_a_ S.D. (µm)
**Peak 1**	1.58	1.09	1.32	1.33	0.25	1.38	0.24
**Peak 2**	1.72	1.20	1.36	1.43	0.27		
**Peak 3**	1.47	1.16	1.54	1.39	0.20		
**Valley 1**	0.94	0.92	0.83	0.90	0.06	0.93	0.10
**Valley 2**	0.88	0.83	0.83	0.85	0.03		
**Valley 3**	0.92	0.94	1.32	1.06	0.23		
